# An ERP Study of the Processing of Common and Decimal Fractions: How Different They Are

**DOI:** 10.1371/journal.pone.0069487

**Published:** 2013-07-24

**Authors:** Li Zhang, Qi Wang, Chongde Lin, Cody Ding, Xinlin Zhou

**Affiliations:** 1 Department of Psychology, Southwest University, Chongqing, China; 2 Institute of Developmental Psychology, Beijing Normal University, Beijing, China; 3 Division of Educational Psychology, Research, and Evaluation, University of Missouri-St. Louis, St. Louis, Missouri, United States of America; 4 State Key Laboratory of Cognitive Neuroscience and Learning, Beijing Normal University, Beijing, China; Cuban Neuroscience Center, Cuba

## Abstract

This study explored event-related potential (ERP) correlates of common fractions (1/5) and decimal fractions (0.2). Thirteen subjects performed a numerical magnitude matching task under two conditions. In the common fraction condition, a nonsymbolic fraction was asked to be judged whether its magnitude matched the magnitude of a common fraction; in the decimal fraction condition, a nonsymbolic fraction was asked to be matched with a decimal fraction. Behavioral results showed significant main effects of condition and numerical distance, but no significant interaction of condition and numerical distance. Electrophysiological data showed that when nonsymbolic fractions were compared to common fractions, they displayed larger N1 and P3 amplitudes than when they were compared to decimal fractions. This finding suggested that the visual identification for nonsymbolic fractions was different under the two conditions, which was not due to perceptual differences but to task demands. For symbolic fractions, the condition effect was observed in the N1 and P3 components, revealing stimulus-specific visual identification processing. The effect of numerical distance as an index of numerical magnitude representation was observed in the P2, N3 and P3 components under the two conditions. However, the topography of the distance effect was different under the two conditions, suggesting stimulus specific semantic processing of common fractions and decimal fractions.

## Introduction

Fractions, the experiential basis for rational numbers, refer to the part-whole concept [1]–[2]. They can be expressed in a nonsymbolic format and a symbolic format. The non-symbolic fraction can also be termed proportion. In this study, we adopt the term “non-symbolic fraction” as used in some previous studies [3]–[4] to emphasize the similarities between non-symbolic and symbolic stimuli. Symbolic fractions, furthermore, can be represented in two forms: common fractions and decimal fractions. The term “common fractions” is denoted by the symbolic form a/b with the denominator and numerator as integers (e.g. 1/2); the term “decimal fractions”, utilizing a decimal point and decimal components, is a base-ten expression of common fractions without the denominator (e.g., 0.5). Each can be transformed into the other, since they connect to the same semantic system. For example, 1/10 = 0.1, which means one share of a whole divided into 10. As such, we aimed to explore whether there was similar numerical representation of common and decimal fractions.

Most previous studies have explored the representation of common and decimal fractions separately. So far, considerable studies have looked into how skilled adults process common fractions as well as how instructors teach fractions and how children learn them [5]–[10]. Among these studies, some have emphasized whether mentally comparing the numerical magnitude of fractions relies on the processing of the whole fraction or merely the processing of the constituent numerator or denominator [11]–[16]. Other studies pay more attention to whether common fractions can be represented on the mental number line [17]–[19]. In addition, a lot of studies explore the processing of decimal fractions in children and adults [20]–[24].

Although considerable studies have explored the representation of common and decimal fractions separately, fewer studies have examined the connections and differences between them. To our knowledge, only two behavioral studies [25]–[26] have compared the processing of common and decimal fractions. One study [25] indicated that decimal fractions and integers were very similar, but common fractions and integers were not. The other study compared the processing of unit fractions and decimal fractions [26]. They found that the distance effect appeared when decimal fractions were compared to integers but not when unit fractions were compared to integers. The distance effect which is usually thought to arise from an ordered representation of the magnitude of numbers on a mental number line [27], refers to the increase of reaction times (RTs) and the decrease of accuracy when the distance between the compared numbers decreases [28]. Therefore, it was concluded that there was an easier mapping of decimal fractions on the same mental number line with whole numbers as compared to unit fractions [26].

Similar to these two studies [25]–[26], this study aimed to compare the processing of common and decimal fractions. However, unlike the two studies, this study used a nonsymbolic and symbolic matching task. An advantage of the matching task is that the distance effect in this task is likely to originate from number representations rather than a decision process. In a sense, our task is actually a same-different judgment task, in which the distance effect has been proved to originate from number representations rather than a decision process [29]–[30]. However, the distance effect in a comparison task does not necessarily indicate the semantic processing [29],[31]. Therefore, the nonsymbolic and symbolic matching task in this study may better reflect the semantic processing of common and decimal fractions. Specifically, the task was divided into two conditions. In the common fraction condition, a nonsymbolic fraction was presented and participants were instructed to compare its magnitude with a subsequent symbolic common fraction. In the decimal fraction condition, participants were instructed to compare a nonsymbolic fraction with a subsequent symbolic decimal fraction.

Our task is reversal to the paradigm used by one previous study [32], where the first stimulus differed in formality and the second was identical. The reason for our design is that the numerical representation may be better investigated with nonsymoblic fractions presented first. In our study, when the second stimulus appeared, participants would be required to accurately respond as soon as possible. As a result, participants are likely to translate the first stimulus in the form identical with the second stimulus since the first stimulus has relatively enough time to be translated. For example, common fractions are probably translated into nonsymbolic fractions if they are presented first, leading to the perceptual matching instead of numerical matching. In addition, it is found that students perform better in translating from nonsymbolic expression (e.g., line segment, number line) to symbolic decimals in comparison to the translation from symbolic decimals to nonsymbolic expression [21]. Therefore, even if nonsymbolic fractions were presented second, participants would probably match nonsymbolic and symbolic fractions by translating nonsymbolic into symbolic fractions, which will complicate the numerical matching processing. In combination with the high temporal resolution event-related potential (ERP) technique, the use of the nonsymbolic and symbolic matching task could allow us to compare the neutral correlates of the processing of common and decimal fractions. We could examine not only the ERP correlates of symbolic common and decimal fractions, but also the influence of task demands on the processing of nonsymbolic fractions by comparing the brain activities elicited by nonsymbolic fractions under the common and decimal fraction conditions. Specifically, with the same nonsymbolic stimuli, this study could explore whether the processing of nonsymbolic fractions when they had to subsequently be compared to common fractions was different from their processing when compared to decimal fractions.

Some studies have argued that number processing can be deconstructed into distinct stages of identification processing and semantic processing [33],[34]. Accordingly, we expected that the identification processing of common and decimal fractions would be different. This is partly because the superficial structures of common and decimal fractions are vastly different. Common fractions have an upper-lower structure with two numbers and a line, whereas decimal fractions have a left-right structure with two numbers and a decimal point. Furthermore, teachers tend to use distinct representational models when teaching common and decimal fractions in actual educational practice [35]. When teaching common fractions, teachers often use area models (e.g., a pie is cut into five equal pieces) and collection models (e.g., one blue ball and four red balls in a group of five balls). When teaching decimal fractions, by contrast, they often use a ruler (1.3 m = 1 m and 30 cm) or money ($1.11 = 1 dollar, 1 dimes, and 1 penny). Thus, the two symbols, though they are based on the same semantic system, receive separate and distinct treatment [35]. These differences in initial teaching and acquisition underlie our prediction that the identification processing of fractions was more likely to be stimulus specific. Based on this line of reasoning, we expected that the processing of nonsymbolic fractions when subsequently compared to common fractions would be different from their processing when compared to decimal fractions. Finally, based on previous studies [25],[26] and similar reasons mentioned above, we hypothesized the semantic processing of common and decimal fractions would be different.

According to previous ERP studies [16],[33],[36]–[41], stimulus identification is usually related to the N1 component, whereas the activation of the magnitude representation affected by the distance effect is mainly reflected by P2, N3 and P3 components. Hence, the current study mainly examined the condition and distance effects in the N1, P2, N3 and P3 components. A significant condition effect in the N1 component would reveal the stimulus-specific visual identification of common and decimal fractions. A significant interaction of condition by distance in the P2, N3 and P3 components would indicate the stimuli-specific semantic processing of common and decimal fractions.

## Methods

### Participants

Fourteen college-educated volunteers participated in the experiment after providing written informed consent. One subject was excluded from data analysis because the amplitude of P3 evoked by symbolic fractions in this subject was extremely big as compared to other subjects after a series of analyses, including baseline correction and artifact rejection. Thus, thirteen subjects remained in the sample (6 females and 7 males aged 19–24 years). All were right handed and had normal or corrected-to-normal vision. They were healthy and had no history of neurological or psychiatric abnormalities. The subjects received financial compensation for their participation. This experiment was approved by the Administration Committee of Psychological Research in Southwest University and was in compliance with the ethical guidelines of the American Psychological Association.

### Task and Stimuli

**Figure 1:**
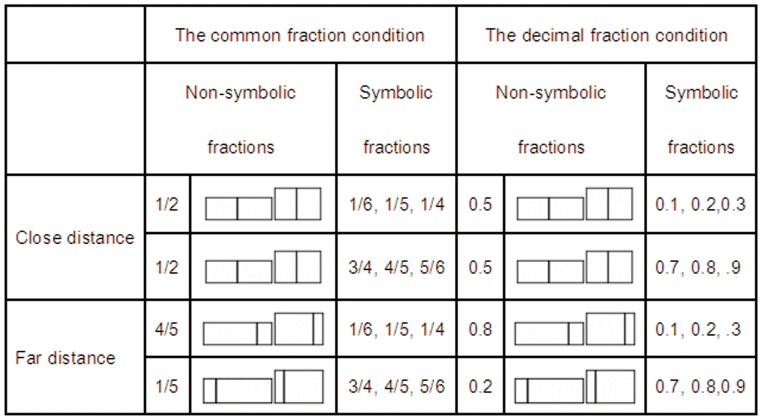
Experimental trials and the corresponding stimuli.

All participants were administered a magnitude matching task under two conditions. In the common fraction condition, participants were presented with a picture which included a bar and a line that divided the bar into two parts, and asked to fixate on and remember the nonsymbolic fraction of the left part of the line to the whole bar. The exact values of these nonsymbolic fractions were 0.2, 0.5, and 0.8, respectively (see Figure 1). Then, a common fraction (1/6, 1/5, 1/4, 2/5, 1/2, 3/5, 3/4, 4/5, or 5/6) was presented and participants were asked to indicate whether the symbolic common fraction and the previous bar represented an equal magnitude. A decimal fraction condition was also performed: a nonsymbolic fraction was presented, followed by a decimal fraction (0.1, 0.2, 0.3, 0.4, 0.5, 0.6, 0.7, 0.8, or 0.9). Under each condition, there were two types of distance which referred to the difference between the magnitude of nonsymbolic and symbolic fractions. The “close” distance was 0.3 on the average, whereas the “far” distance was 0.6 on the average.

In order to prevent participants from concentrating on a specific desirable size of nonsymbolic fractions and on a specific desirable relationship between nonsymbolic and symbolic fractions, a total of 54 trials (3 nonsymbolic fractions×2 sizes for nonsymbolic fractions×9 symbolic fractions) were designed under each condition. All these trials were repeated 8 times, so the task under each condition consisted of 432 trials. Among these trials, a total of 192 experimental trials were used and analyzed: 96 trials for the far distance and 96 trials for the close distance. These trials and stimuli are showed in Figure 1. The remaining 240 trials were used as the control trials and not analyzed.

All stimuli were presented in black on a gray background and subtended a visual angle of less than 6°. Nonsymbolic fractions denoted by bars had two sizes: 3.9°×2.7° and 5.5°×2° with an equal area approximately 12 cm2. Common fractions were 0.9°×1.9° presented in vertical form and decimal fractions were 1.5°×1°. The viewing distance was about 60 cm.

### Procedure

Each subject was seated in a comfortable armchair in a dimly lit and sound-attenuated room. All participants completed two conditions. One was the common fraction condition; the other was the decimal fraction condition. The order of conditions was counterbalanced across participants. For each condition, participants were asked to focus on the center of the screen with their left and right hands placed on the “F” and “J” keys respectively. They were instructed to press the “F” key with the forefinger of left hands if the two numerals represented the same magnitude and the “J” key with the forefinger of right hands if they represented different magnitudes. Instructions emphasized both speed and accuracy.

Each trial started with a blank bar in the middle of the screen for 500 ms. Then, the bar with a separate line was displayed in the center for 1000 ms, which was cleared by a blank screen for a random duration of 800–1000 ms. After the screen was cleared, the fraction number was displayed until a response was recorded (maximum 1500 ms). After an interval of 1500 ms, the next trial would begin. The whole procedure was controlled by E-prime 1.1.

There were 16 practice trials excluded from the analysis in each condition before recording commenced. Participants were permitted into the formal experimental session when their accumulated accuracy reached more than 80%. Each experimental condition was divided into 4 blocks, with each block 108 trials. After each block, participants were allowed to take a break and proceed to the next block at his/her own pace. The whole experiment run-time lasted approximately 60 minutes per subject. In addition, each condition was presented pseudo-randomly across stimuli to make sure no repetition of stimulus on consecutive trials.

### Electrophysiology

Electroencephalography (EEG) was recorded from 64-channel scalp sites using tin electrodes mounted in an elastic cap (Brain Products, GmbH, Germany), with the average references on the left and right mastoids and a ground electrode situated on the middle of the forehead (AFz). Horizontal and vertical electrooculograms (EOGs) were also recorded. All inter-electrode impedances were maintained below 5 kΩ during recording. EEGs were recorded continuously with a 0.1–40 Hz bandpass and continuously sampled at 500 Hz/channel for signal amplification. The EEG data were processed offline to reject trials with EOG artifacts (mean EOG voltage exceeding ±80 µV), eye movement, blinking, motion, and other artifacts at any of the channels. Grand-average ERPs were corrected on the 100-ms pre-stimulus baseline and low-pass filtered at 40 Hz. There were two segments of ERP recording which were time-locked to the onset of the bar denoting a nonsymbolic fraction and to the onset of the symbolic fraction.

In order to adequately demonstrate the processing of common and decimal fractions in the brain, we selected 15 widely distributed and representative electrodes located in five regions for analysis: F3, Fz, F4 (frontal region), C3, Cz, C4 (central region), P3, Pz, P4 (parietal region), PO3, POz, PO4 (parieto–occipital region), O1, Oz and O2 (occipital region). For nonsymbolic fractions, the N1, P2, and P3 components were analyzed. The peak latency and mean amplitude of the N1, P2 and P3 were measured between 120–180 ms, between 180–240 ms, and between 240–340 ms, respectively. Through visual inspection of the average waveforms and guided by previous studies [32]–[34],[41], the post-stimulus time windows for the major ERP components evoked by symbolic fractions were as follows: 100–180 ms (N1), 180–260 ms (P2), 260–360 ms (N3), and 360–560 ms (P3). We measured the peak latency of each component and the mean amplitude of the N1 between 100–180 ms, the P2 between 180–260 ms, and the P3 between 360–460 ms and 460–560 ms.

## Results

### Behavioral Results

**Figure 2:**
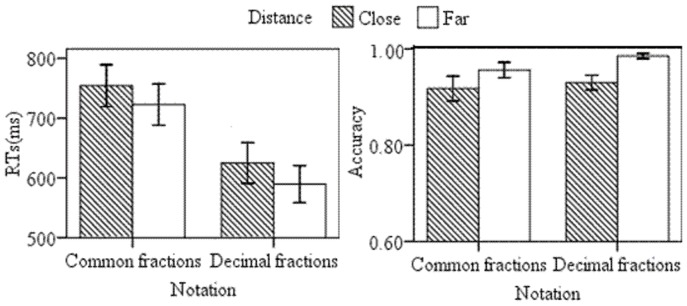
The mean RTs and accuracy of all participants as a function of numerical notation and distance.

Incorrect trials and trials with RTs shorter than 200 ms (5.41%) were excluded for RTs analyses. For each participant, the mean RTs for the far and close distances in the common and decimal fraction conditions were calculated. The behavioral data are summarized in Figure 2. Our analysis of RTs revealed a main effect for condition, *F*(1, 12) = 36.04, *p = *0.000. The mean RTs in the common fraction condition were 131 ms slower than that in the decimal fraction condition. There was a main effect of numerical distance, *F*(1, 12) = 21.68, *p = *0.001, with the mean RTs of the close distance being 34 ms slower than that of the far distance. No significant interaction of numerical distance×condition was observed (*p = *0.613).

Analysis of accuracy rates revealed a main distance effect with higher accuracy for the far distance than for the close distance, *F*(1, 12) = 5.61, *p = *0.035. There was no significant main effect of notation and no significant interaction.

### Electrophysiological Results

#### The Processing of Nonsymbolic Fractions

**Figure 3:**
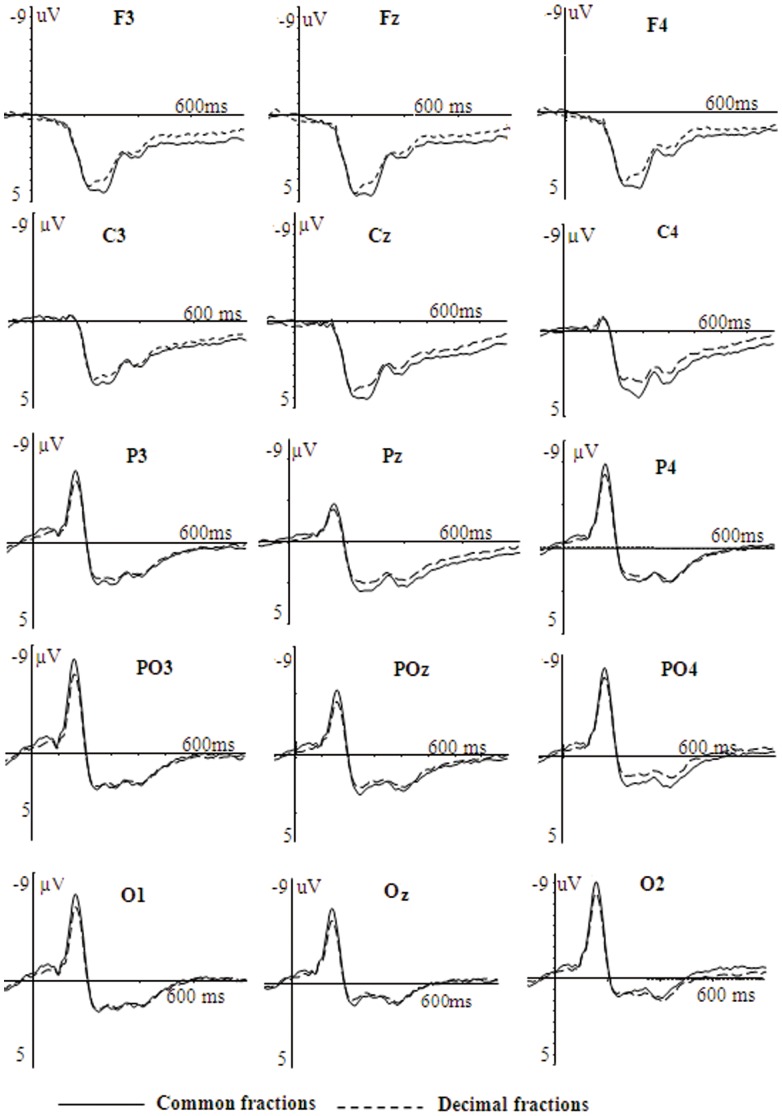
The grand average ERPs of non-symbolic fractions in the common and decimal fraction conditions.

The ERP waveforms evoked by nonsymbolic fractions in the two conditions are shown in Figure 3. Repeated measures analyses of variance (ANOVAs) with condition (common fractions vs. decimal fractions) and hemisphere (left, midline, right) as two within-subject variables were conducted on the amplitudes and latencies of N1, P2 and P3 in each region using Greenhouse-Geisser corrected degrees of freedom for the F-ratio.

On the mean amplitude of the N1, the condition effect was significant at the parietal and occipital sites, *F*s>4.77, the higher *p* was 0.050, with common fractions eliciting greater negativity than decimal fractions (parietal: −4.58 µV vs. −3.94 µV; occipital: −6.17 µV vs. −5.25 µV). At parieto-occipital sites, the interaction of condition×hemisphere was significant, *F*(1, 12) = 5.57, *p* = 0.010. Follow-up analyses showed that the interaction was due to significant condition effect over PO3 and POz, *F*s>5.42, the higher *p* was 0.038, but not over PO4 (*p* = 0.175). At frontal and central sites, the condition effect was not significant (*F*s<1). On the peak latency of the N1, no significant condition effect was found across all the regions.

On the mean amplitude and peak latency of the P2, no significant condition effect was found across all the regions. On the mean amplitude of the P3, the condition effect was marginally significant at the central sites, *F*(1, 12) = 4.36, *p* = 0.059, with common fractions eliciting greater positivity than decimal fractions (5.63 µV vs. 4.83 µV). In the other four regions, no significant condition effect was found. On the peak latency of the P3, no significant condition effect was observed across all the regions.

**Figure 4:**
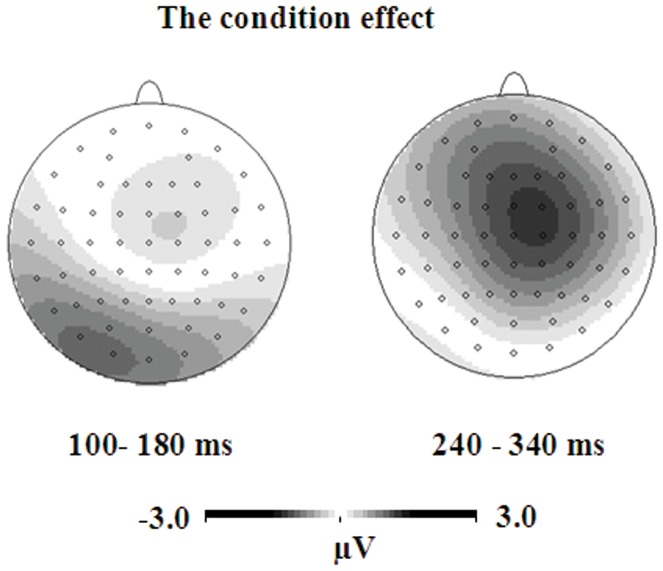
The topographical maps of the condition effect for the nonsymbolic fractions.

The topographic maps of the condition effect are presented in Figure 4. We could see that the condition effect was associated with the amplitude of N1 over left parieto-occipital electrodes. In addition, the condition effect also appeared over central electrodes between 240–340 ms.

#### The Processing of Symbolic Fractions

**Figure 5:**
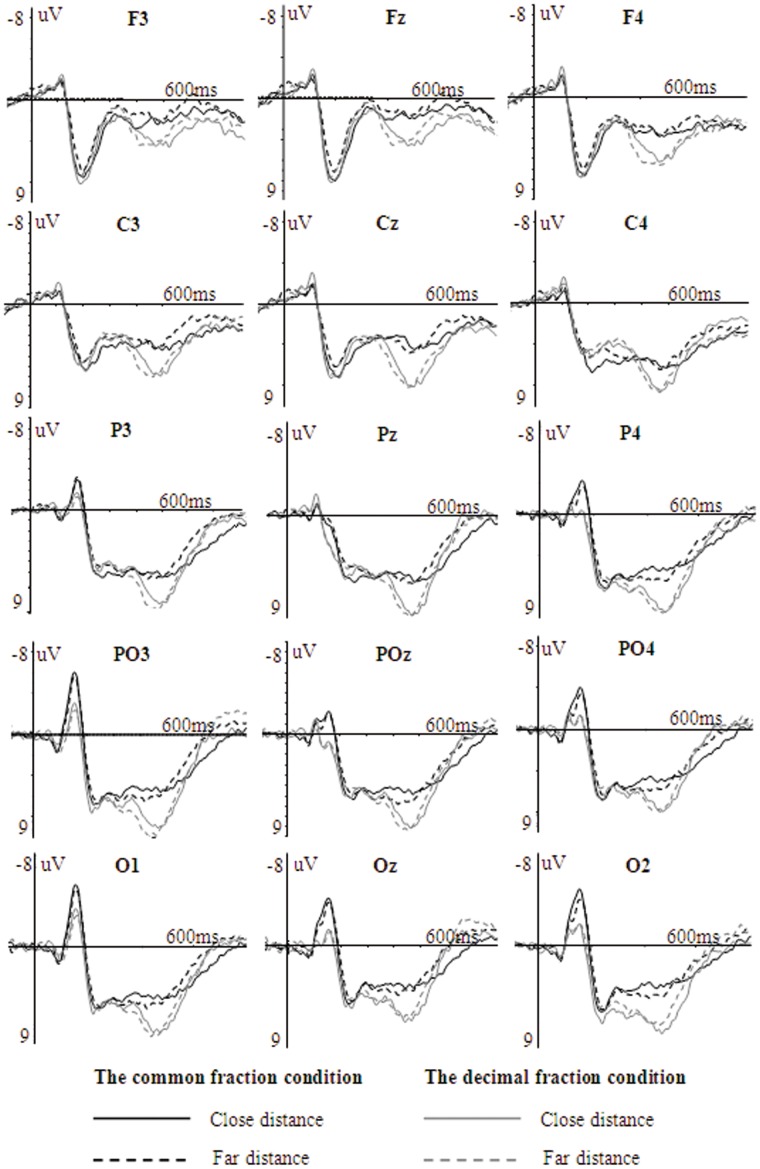
The grand average ERPs evoked by symbolic fractions in the common and decimal fraction conditions.

The ERP waveforms evoked by symbolic fractions as a function of notation and distance are shown in Figure 5. Repeated measures ANOVAs with condition (common fractions vs. decimal fractions), distance (close vs. far), and hemisphere (left, midline, right) as three within-subject variables were conducted on the amplitudes and latencies of N1, P2, N3 and P3 in each region, using Greenhouse-Geisser corrected degrees of freedom for the F-ratio.

On the mean amplitude of the N1, the main effect of condition was found at the parieto-occipital and occipital sites, *F*s>11.42, the higher *p* was 0.003, with common fractions eliciting greater negativity than decimal fractions (parieto-occipital: −2.26 µV vs. −0.32 µV; occipital: −3.14 µV vs. −1.06 µV). At parietal sites, an interaction of condition×distance×hemisphere was observed, *F*(2, 24) = 5.44, *p* = 0.011. The follow-up analyses showed the interaction was due to a significant condition effect observed for the far distance over P3, Pz, P4 and for the close distance over P4 (*F*s>5.51, the highest *p* was 0.037). At frontal and central sites, no relevant effects were found. On the peak latency of the N1, the significant condition effect was observed in the parietal and parieto-occipital sites, *F*s>6.73, the higher *p* was 0.023, with longer latency for common fractions than for decimal fractions (parietal: 137 ms vs. 128 ms; parieto-occipital: 145 ms vs. 136 ms). At frontal, central and occipital sites, no relevant effect was observed.

**Figure 6:**
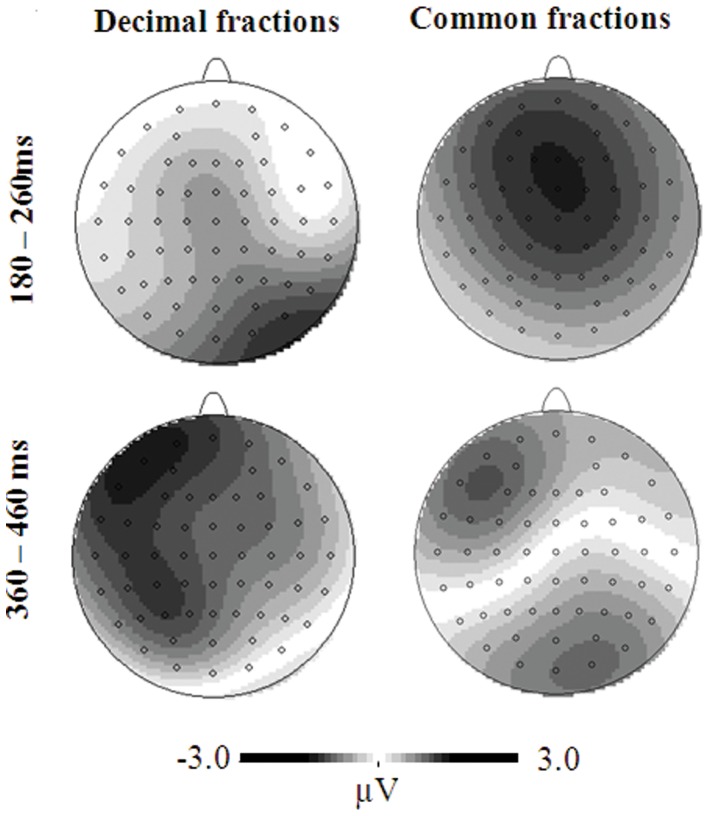
The topographical maps of the distance effect for both common and decimal fractions.

On the mean amplitude of the P2, the distance effect was observed in all the five regions, *F*s>5.16, the highest *p* was 0.042, with the close distance more positive than the far distance(frontal: 6.14 µV vs. 5.20 µV; central: 5.70 µV vs. 4.84 µV; parietal: 5.30 µV vs. 4.46 µV; parieto-occipital: 5.08 µV vs. 4.35 µV; occipital: 4.59 µV vs. 3.86 µV). On the peak latency of the P2, the distance effect was observed at occipital sites, *F*(1, 12) = 7.33, *p* = 0.019, with earlier latency for the close distance than for the far distance 232 ms vs. 239 ms. At other sites, no relevant effect was observed. Although no interaction of condition×distance was observed on the amplitude and latency of P2, the distance effect for common fractions was obvious over fronto-central electrodes but the distance effect for decimal fractions was obvious over parieto-occipital electrodes between 180–260 ms, as seen in Figure 6.

On the mean amplitude of the N3, no relevant effect was found. On the peak latency of the N3, the distance effect was only significant at the occipital sites, *F*(1, 12) = 5.99, *p* = 0.031, with longer latency for the close distance (309 ms) than for the far distance (301 ms).

On the mean amplitude of the P3 between 360–460 ms, the condition effect was observed in the frontal, parietal, parieto-occipital and occipital regions, *F*s>5.36, the highest *p* was 0.039, with more positivity for decimal fractions than for common fractions(frontal: 4.12 µV vs. 2.21 µV; parietal: 8.01 µV vs. 5.93 µV; parieto-occipital: 7.63 µVvs. 5.61 µV; occipital: 6.91 µV vs. 4.54 µV). At central sites, the condition effect was marginally significant, *F*(1, 12) = 4.13, *p* = 0.065, with larger amplitude for decimal fractions (5.85 µV) than common fractions (4.18 µV). In addition, at parieto-occipital sites, the distance effect was also significant, *F*(1, 12) = 10.37, *p* = 0.007, with the far distance (7.07 µV) more positive than the close distance (6.17 µV). Although no interaction of condition×distance was observed, the distance effect over the occipital sites between 360–460 ms was obvious for common fractions as compared to that for decimal fractions, as seen from the topography in Figure 6.

On the mean amplitude of the P3 between 460–560 ms, the condition effect was observed in the parietal and occipital regions, *F*s>5.05, the higher p was 0.044, with more positivity for decimal fractions than for common fractions (parietal:7.77 µV vs. 5.58; occipital: 6.00 µV vs. 3.86 µV). At the parieto-occipital sites, the condition effect was marginally significant, *F*(1, 12) = 4.62, 0.053, with larger amplitude for decimal fractions (6.96 µV) than for common fractions (4.95 µV). No distance effect was found on the mean amplitude of the P3 between 460–560 ms.

**Figure 7:**
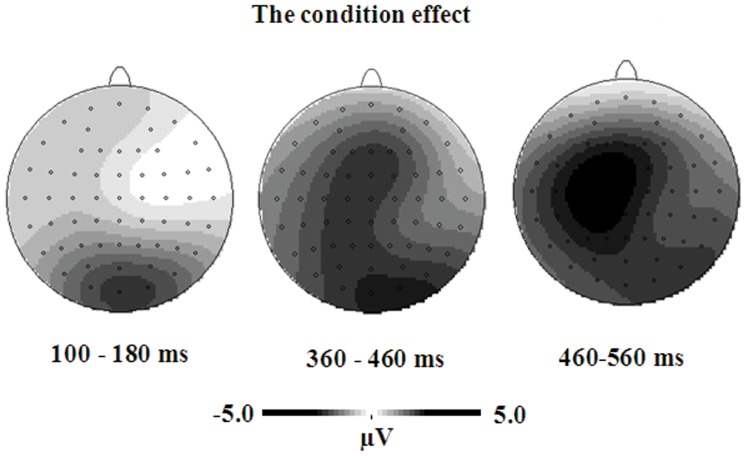
The topographical maps of the condition effect for symbolic fractions.

On the peak latency of the P3, the distance effect was significant at the central, parietal, parieto-occipital and occipital sites, *F*s>4.91, the highest *p* was 0.047, with earlier latency for the far distance than for the close distance (central: 451 ms vs. 473 ms; parietal: 441 ms vs. 461 ms; parieto-occipital: 434 ms vs. 457 ms; occipital: 434 ms vs. 457 ms). No condition effect was found. Figure 7 shows the topographic maps of the condition effect. The condition effect was obvious over parieto-occipital electrodes between 100–180 ms and over widely distributed central, parietal, and occipital electrodes between 360–560 ms.

## Discussion

This study was the first to explore similarities and differences in the processing of common and decimal fractions using ERP technique. As expected, the condition effect was observed in the analyses of both behavioral and electrophysiological data, suggesting that the visual identification of fractions were different in the common and decimal conditions. First, the task specific identification was revealed in the N1 and P3 components evoked by nonsymbolic fractions. When nonsymbolic fractions were compared to common fractions, they displayed larger amplitudes than when they were compared to decimal fractions. Second, the stimulus specific identification was observed in the N1 and P3 components elicited by symbolic fractions with larger N1 amplitude, longer N1 latency, smaller P3 amplitude, and shorter P3 latency for common fractions as compared to decimal fractions. In addition, participants responded slower in the common fraction condition than in the decimal fraction condition, indicating different processing in the common and decimal fraction conditions.

A significant distance effect was found, but no interaction was observed between condition and distance in either behavioral or electrophysiological analyses. This suggested that the semantic processing of common and decimal fractions may be similar. However, the topography of the distance effect revealed some differences in the semantic processing of common and decimal fractions. The semantic processing occurred over fronto-central electrodes for common fractions but over parieto-occipital electrodes for decimal fractions during the time window of P2. In addition, during the time window of P3 the distance effect of common fractions tended to trigger occipital activation as compared to that of decimal fractions.

Taken together, the findings suggest that the visual identification of common and decimal fractions were different. However, there were both similarities and differences in the semantic processing of common and decimal fractions.

### The Processing of Nonsymbolic Fractions

In this study, the condition effect was mainly indicated by the N1 component in a parieto-occipital network, characterized by larger amplitude for common fractions compared with decimal fractions. Differences in the N1 component have been attributed to high-level visual identification processing, including sensory processing or perceptual load [38]. Consistent with previous research on integers [33]–[34],[36],[42], therefore, the finding of the current study suggests that the identification processing of nonsymbolic fractions is task dependent.

Notably, the fact that the same nonsymbolic fractions were presented in both the common and decimal fraction conditions suggests that the condition effect observed on the N1 component was not due to perceptual differences but due to task demands. This suggests that there are considerable differences between the processing of common and decimal fractions. Even when symbolic fractions were not presented, visual processing began to differ due to different task demands. We speculated that participants adopted distinct mental representations influenced by task demands. For instance, they might have used an area model for common fractions and a ruler model for decimal fractions. As stated previously, area models are often used for teaching common fractions and ruler models for decimal fractions, and this may have affected participants’ mental representation and processing of common and decimal fractions. An area model requires more attention and wider sensory processing, which may have led to the larger amplitudes for the common fraction condition.

Another possibility is that in the decimal fraction condition participants tend to divide the non-symbolic fraction into 10 equal pieces because decimal fractions are based on ten. In contrast, this strategy is less possible in the common fraction condition because the denominator is always changing. A participant will not necessarily divide the non-symbolic fraction into 10 parts as done in the decimal fraction condition. For example, the non-symbolic fraction 1/2 may be divided into 2 pieces and 1/5 may be divided into 5 pieces. The number of pieces that the non-symbolic fraction is divided into is likely to be variable from trial to trial. The non-symbolic fraction then relatively requires more attention in the common fraction condition, resulting in a larger N1 for the common fraction condition than for the decimal fraction condition. In future studies, this explanation can be tested by using only common fractions with the same denominator (2/10, 3/10…….).

In addition, nonsymbolic fractions under the common fraction condition evoked greater P3 than those under the decimal fraction condition at the central sites, which may be due to the fact that nonsymbolic fractions under the common fraction condition require more attention. In this study, the P3 evoked by nonsymbolic fractions is expected to be an early attention process, which can be termed the P3a. The attention-driven P3a usually has a central maximum amplitude distribution and relatively short peak latency [43]. Indeed, the P3 is found more obvious over frontal and central sites based on the grand average ERPs evoked by nonsymbolic fractions (see Figure 3), and the topographical map for the condition effect revealed that the common and decimal fraction conditions differed over the central sites between 240-340 ms (see Figure 4). The P3a is different from the P3 evoked by symbolic fractions, which is longer in its latency and more obvious over posterior parieto-occipital sites (see Figure 5).

### The Processing of Symbolic Fractions

When participants judged whether the numerical magnitude of symbolic and nonsymbolic fractions matched, we observed a condition effect in the N1 amplitude and latency, indicating that the visual identification of common and decimal fractions was different. It is thought that the amplitude of the posterior N1 reflects a visual discriminative process that is applied to attended stimuli, and that the latency of N1 may reflect the time course of discriminative processing [44]. In addition, it has been argued that the N1 latency reflects the attentional effort required for visual processing as latency seems to increase during tasks that are significantly complex [45]. Therefore, the larger N1 amplitude and longer N1 latency observed for common fractions compared with decimal fractions in the present study reflected the fact that the identification of common fractions was more complex and necessitated longer discriminative processing and more discriminative attention. This interpretation is in line with the behavioral findings which showed that decimal fractions were processed faster than common fractions. Indeed, previous studies have found that children have more difficulty with common fractions [46]. The relative difficulty may stem from the reduced exposure to common fractions following the introduction, and subsequent widespread use of decimal fractions.

The condition effect was also found in the P3 component, wherein larger amplitude was observed for decimal fractions. In previous studies, the P3 amplitude has been associated with motor preparation and execution, and response confidence [33] or cognitive load [47]. Larger P3 amplitudes might reflect a more forceful and confident response for decimal fractions compared to common fractions or less cognitive load for decimal fractions compared to common fractions.

Consistent with previous ERP studies [33],[37]–[38],[40]–[41], we detected a distance effect in the P2, N3, and P3 components. The close distances had larger P2 amplitudes and shorter P2 latencies when compared to far distances, and a reversal trend was observed in the polarity of distance effect in the P3 amplitude and latency as well as the N3 latency. In addition, the distance effect was not restricted to the parietal electrodes, but it was seen over widely distributed frontal, central, and parietal electrodes, as seen in some studies [40],[48]. Most importantly, consistent with behavioral results, no interaction was demonstrated between distance and notation in each ERP component, which seemed to indicate that the numerical representation of common and decimal fractions was similar.

However, as seen in Figure 6, the semantic processing of common fractions involved a fronto-central network between 180–260 ms, but decimal fractions involved a parieto-occipital network. This finding is consistent with previous two imaging studies [13],[49], which revealed frontal activations in the representation of fractions in the form of a/b. A possible explanation may be that the numerical magnitude of common fractions is usually complex relative to decimal fractions. Except for some common fractions frequently used (e.g., 1/2, 1/3, 1/4), a learned mapping between fractions and mental magnitudes seems implausible because there are an infinite number of equivalent fractions for a real value [14]. Therefore, the real value of a common fraction is probably available through accessing the magnitude of its components, followed by a division operation. In contrast, the numerical access of decimal fractions does not involve such procedures. As a result, the representation of common fractions involved more frontal activations, which are usually found in complex calculations [33],[50]–[51].

In addition, during the time window of P3 the distance effect of common fractions tended to trigger occipital activation as compared to that of decimal fractions. A possible reason may be that participants matched nonsymbolic fractions and common fractions by translating common fractions into nonsymbolic fractions. Fractions written in the symbolic form a/b are closely associated with the part-whole relationship [2]. Using base-10 notation to represent fractional quantities, decimal fraction have many of the same features as the notation used to represent whole-number quantities, leading to more understanding of decimal fractions as static units, not the part-whole relationship [52]. Moreover, area models as used in this study are usually adopted for the teaching of common fractions. Therefore, it may be much easy to translate common fractions into nonsymbolic area models, which emphasizes the part-whole relationship. Matching two nonsymbolic fractions would depend on occipital sites that are usually associated with visual and perceptual processing.

### Conclusion

The present study was the first to examine the ERP correlates of the processing of common and decimal fractions using the numerical magnitude matching task. The results showed that the visual identification processing of common and decimal fractions was distinct. The semantic magnitude processing of common and decimal fractions was similar, based on analyses of the amplitudes and latencies of ERP components. However, the stimulus specific topographic maps of ERPs revealed some differences in the semantic processing of common and decimal fractions. Further studies with higher spatial resolution technique such as functional magnetic resonance imaging are needed to investigate the brain organization for the semantic processing of common and decimal fractions.

## References

[1] Behr M, Lesh R, Post T, Silver E (1983) Rational number concepts. In R. Lesh & M. Landau (Eds.), Acquisition of mathematics concepts and processes (pp. 91–125) New York: Academic Press.

[2] Ni YJ, Zhou YD (2005) Teaching and learning fraction and rational numbers: The origins and implications of whole number bias. Educational Psychologist 40(1): 27–52.

[3] Siegler RS, Fazio LK, Bailey DH, Zhou X (2013) Fractions: The new frontier for theories of numerical development. Trends in Cognitive Science 17: 13–19. 10.1016/j.tics.2012.11.004 23219805

[4] Fazio LK, Bailey DH, Thompson CA, Siegler RS (2013, Apr). Relations of symbolic and non-symbolic fraction and whole number magnitude representations to each other and to mathematics achievement. Spoken presentation to be given at the biennial meeting of the Society for Research on Child Development, Seattle, WA.

[5] Bright GW, Behr MJ, Post TR, Wachsmuth I (1988) Identifying fractions on number lines. Journal for Research in Mathematics Education 19: 215–232.

[6] Gabriel F, Coche F, Szűcs D, Carette V, Rey B, et al (2012) An intervention program to improve children’s understanding of fractions. Mind, Brain and Education 6(3): 137–146.

[7] Hecht SA, Vagi KJ, Torgesen JK (2007) Fraction skills and proportional reasoning. In D. B. Berch & M. M. M. Mazzocco (Eds.), Why is math so hard for some children? The nature and origins of mathematical learning difficulties and disabilities (pp. 121–132). Baltimore: Paul H. Brookes Publishing Co.

[8] Mack N (1995) Confounding whole-number and fraction concepts when building on informal knowledge. Journal for Research in Mathematics Education 26: 422–441.

[9] Ni YJ (2001) Semantic domains of rational numbers and the acquisition of fraction equivalence. Contemporary Educational Psychology 26: 400–417. 11414728 10.1006/ceps.2000.1072

[10] Smith CL, Solomon GEA, Carey S (2005) Never getting to zero: Elementary school students’ understanding of the infinite divisibility of number and matter. Cognitive Psychology 51(2): 101–140. 16081058 10.1016/j.cogpsych.2005.03.001

[11] Bonato M, Fabbri S, Umiltà C, Zorzi M (2007) The mental representation of numerical fractions, real or integer? Journal of Experimental Psychology: Human Perception and Performance 33: 1410–1419. 18085953 10.1037/0096-1523.33.6.1410

[12] Faulkenberry TJ, Pierce BH (2011) Mental representations in fraction comparison: Holistic versus component-based strategies. Experimental Psychology 58: 480–489. 21592948 10.1027/1618-3169/a000116

[13] Ischebeck A, Schocke M, Delazer M (2009) The processing and representation of fractions within the brain: An fMRI investigation. NeuroImage 47: 403–413. 19328235 10.1016/j.neuroimage.2009.03.041

[14] Meert G, Grégoire J, Noël MP (2009) Rational numbers: Componential versus holistic representation of fractions in a magnitude comparison task. Quaterly Journal of Experimental Psychology 62(8): 1598–1616. 10.1080/17470210802511162 19123119

[15] Sprute LA, Temple E (2011) Representations of fractions: evidence for accessing the whole magnitude in adults. Mind, Brain, and Education 5: 42–47.

[16] Zhang L, Xin ZQ, Li FH, Wang Q, Ding C, et al (2012) An ERP study on the processing of common fractions. Experimental Brain Research 217: 25–34. 22159550 10.1007/s00221-011-2969-4

[17] Ganor-Stern D (2012) Fractions but not negative numbers are represented on the mental number line. Acta Psychologica 139: 350–357. 22192439 10.1016/j.actpsy.2011.11.008

[18] Kallai AY, Tzelgov J (2009) A generalized fraction: An entity smaller than one on the mental number line. Journal of Experimental Psychology: Human Perception and Performance 35: 1845–1864. 19968440 10.1037/a0016892

[19] Siegler RS, Thompson CA, Schneider M (2011) An integrated theory of whole number and fractions development. Cognitive Psychology 62(4): 273–296. 21569877 10.1016/j.cogpsych.2011.03.001

[20] Cohen DJ (2010) Evidence for direct retrieval of relative quantity information in a quantity judgment task: decimals, integers, and the role of physical similarity. Journal of Experimental Psychology: Learning, Memory, and Cognition 36: 1389–1398. 10.1037/a0020212 PMC2970732 20804282

[21] Michaelidou N, Gagatsis A, Pitta-Pantazi D (2004) The number line as a representation of decimal numbers: A research with sixth grade students. In M. J. Hoines & A. B. Fuglestad (Eds.), Proceedings of the 28th Conference of the International Group for the Psychology of Mathematics Education (Vol. 3, 305–312). Bergen, Norway: PME.

[22] Rittle-Johnson B, Siegler RS (2001) Developing Conceptual Understanding and Procedural Skill in Mathematics: An Iterative Process. Journal of Educational Psychology 93(2): 346–362.

[23] Steinle V, Stacey K (2004) A longitudinal study of students' understanding of decimal notation: An overview and refined results. In I. Putt, R. Faragher & M. McLean (Eds.), Proceedings of the 27th Annual Conference of the Mathematics Education Research Group of Australasia (Vol. 2, 541–548). Townsville: MERGA.

[24] Varma S, Karl SR (2013) Understanding decimal proportions: Discrete representations, parallel access, and privileged processing of zero. Cognitive Psychology 66(3): 283–301. 23416180 10.1016/j.cogpsych.2013.01.002

[25] Iuculano T, Butterworth B (2011) Understanding the real value of fractions and decimals. The Quarterly Journal of Experimental Psychology 64(11): 2088–2098. 21929473 10.1080/17470218.2011.604785

[26] Ganor-Stern D (2013) Are 1/2 and 0.5 represented in the same way? Acta Psychologica 142(3): 299–307. 23419807 10.1016/j.actpsy.2013.01.003

[27] Dehaene S (1997) The number sense. Oxford University Press, New York.

[28] Moyer RS, Landauer TK (1967) Time required for judgments of numerical inequality. Nature 215: 1519–1520. 6052760 10.1038/2151519a0

[29] Van Opstal F, Gevers W, De Moor W, Verguts T (2008) Dissecting the symbolic distance effect: Priming and comparison distance effects in numerical and non-numerical orders. Psychonomic Bulletin & Review 15(2): 419–425. 18488662 10.3758/pbr.15.2.419

[30] Van Opstal F, Verguts T (2011) The origins of the numerical distance effect: The same-different task. Journal of Cognitive Psychology 23(1): 112–120.

[31] Cohen Kadosh R, Brodsky W, Levin M, Henik A (2008) Mental representation: What can pitch tell us about the distance effect? Cortex 44: 470–477. 18387580 10.1016/j.cortex.2007.08.002

[32] Szűcs D, Csépe V (2004) Access to numerical information is dependent on the modality of stimulus presentation in mental addition: A combined ERP and behavioral study. Cognitive Brain Research 19: 10–27. 14972354 10.1016/j.cogbrainres.2003.11.002

[33] Dehaene S (1996) The organization of brain activations in number comparison: Event-related potentials and the additive-factors method. Journal of Cognitive Neuroscience 8: 47–68. 23972235 10.1162/jocn.1996.8.1.47

[34] Pinel P, Dehaene S, Rivière D, LeBihan D (2001) Modulation of parietal activation by semantic distance in a number comparison task. NeuroImage 14: 1013–1026. 11697933 10.1006/nimg.2001.0913

[35] Lachance A, Confrey J (2002) Helping students build a path of understanding from ratio and proportion to decimal symbol. The Journal of Mathematical Behavior 20: 503–526.

[36] Cao BH, Li FH, Li H (2010) Notation-dependent processing of numerical magnitude: electrophysiological evidence from Chinese numerals. Biological Psychology 83, 47–55. 10.1016/j.biopsycho.2009.10.003 19854237

[37] Jiang T, Qiao S, Li J, Cao Z, Gao X, et al (2010) Effects of symbol type and numerical distance on the human event-related potential. Neuropsychologia 48: 201–210. 19751750 10.1016/j.neuropsychologia.2009.09.005

[38] Libertus M, Woldorff M, Brannon E (2007) Electrophysiological evidence for notation independence in numerical processing. Behavioral and Brain Functions 3(1): 1–15. 17214890 10.1186/1744-9081-3-1 PMC1781950

[39] Szűcs D, Csépe V (2004) Similarities and differences in the coding of numerical and alphabetical order using acoustic stimulation as revealed by event-related potentials in humans. Neuroscience Letters 360: 65–68. 15082180 10.1016/j.neulet.2004.02.038

[40] Szűcs D, Csépe V (2005) The effect of numerical distance and stimulus probability on ERP components elicited by numerical incongruencies in mental addition. Cognitive Brain Research 22: 282–300. 10.1016/j.cogbrainres.2004.04.010 15653300

[41] Temple E, Posner M (1998) Brain mechanisms of quantity are similar in 5-year-old children and adults. Proceeding of the National Academy of Sciences (USA) 95: 7836–7841. 10.1073/pnas.95.13.7836 PMC22775 9636237

[42] Plodowski A, Swainson R, Jackson GM, Rorden C, Jackson SR (2003) Mental representation of number in different numerical forms. Current Biology 13: 2045–2050. 14653993 10.1016/j.cub.2003.11.023

[43] Polich J (2007) Updating P300: An integrative theory of P3a and P3b. Clinical Neurophysiology 118: 2128–2148. 17573239 10.1016/j.clinph.2007.04.019 PMC2715154

[44] Vogel EK, Luck SJ (2000) The visual N1 component as an index of a discrimination process. Psychophysiology 37: 190–203. 10731769

[45] Callaway E, Halliday R (1982) The effect of attentional effort on visual evoked potential N1 latency. Psychiatry Research 7: 299–308. 6962438 10.1016/0165-1781(82)90066-x

[46] Brousseau G, Brousseau N, Warfield V (2004) Rationals and decimals as required in the school curriculum Part 1: Rationals as measurement. The Journal of Mathematical Behavior 23: 1–20.

[47] Kok A (2001) On the utility of P3 amplitude as a measure of processing capacity. Psychophysiology 38(3): 557–577. 11352145 10.1017/s0048577201990559

[48] Zhou X, Chen C, Dong Q, Zhang H, Chen C, et al (2006) Numerical distance effect in the N240 component in a number-matching task. Neuroreport 17(10): 991–994. 16791090 10.1097/01.wnr.0000221840.12632.9f

[49] Jacob SN, Nieder A (2009) Notation-independent representation of fractions in the human parietal cortex. Journal of Neuroscience 29: 4652–4657. 19357289 10.1523/JNEUROSCI.0651-09.2009 PMC6665727

[50] Gruber O, Indefrey P, Steinmetz H, Kleinschmidt A (2001) Dissociating neural correlates of cognitive components in mental calculation. Cereb Cortex 11: 350–359. 11278198 10.1093/cercor/11.4.350

[51] Prabhakaran V, Rypma B, Garbrieli JD (2001) Neural substrates of mathematical reasoning: a functional magnetic resonance imaging study of neocortical activation during performance of the necessary arithmetic operations tests. Neuropsychology 15: 115–127. 11216882 10.1037//0894-4105.15.1.115

[52] Irwin KC, Britt MS (2004) Operating with decimal fractions as a part-whole concept. In I. Putt, R. Faragher & M. McLean (Eds.), Mathematics education for the third millennium: Towards 2010 (Proceedings of the 27th annual conference of the Mathematics Education Group of Australasia, 312–319). Sydney: MERGA.

